# Granger Causality Mapping during Joint Actions Reveals Evidence for Forward Models That Could Overcome Sensory-Motor Delays

**DOI:** 10.1371/journal.pone.0013507

**Published:** 2010-10-21

**Authors:** Idil Kokal, Christian Keysers

**Affiliations:** 1 Department of Neuroscience, BCN Neuroimaging Centre, University Medical Center Groningen, Groningen, The Netherlands; 2 The Netherlands Institute for Neuroscience, Royal Netherlands Academy of Arts and Sciences (KNAW), Amsterdam, The Netherlands; The University of Western Ontario, Canada

## Abstract

Studies investigating joint actions have suggested a central role for the putative mirror neuron system (pMNS) because of the close link between perception and action provided by these brain regions [1], [2], [3]. In contrast, our previous functional magnetic resonance imaging (fMRI) experiment demonstrated that the BOLD response of the pMNS does not suggest that it directly *integrates* observed and executed actions during joint actions [4]. To test whether the pMNS might contribute indirectly to the integration process by sending information to brain areas responsible for this *integration* (integration network), here we used Granger causality mapping (GCM) [5]. We explored the directional information flow between the anterior sites of the pMNS and previously identified integrative brain regions. We found that the left BA44 sent more information than it received to both the integration network (left thalamus, right middle occipital gyrus and cerebellum) and more posterior nodes of the pMNS (BA2). Thus, during joint actions, two anatomically separate networks therefore seem effectively connected and the information flow is predominantly from anterior to posterior areas of the brain. These findings suggest that the pMNS is involved indirectly in joint actions by transforming observed and executed actions into a common code and is part of a generative model that could predict the future somatosensory and visual consequences of observed and executed actions in order to overcome otherwise inevitable neural delays.

## Introduction

Joint action is defined as the coordination of the actions of two or more individuals in time and space in order to bring about a change in the environment [6]. The neural circuitry behind joint actions has recently been investigated in a number of fMRI studies [2], [4], [7], [8]. Most of these studies [2], [7], [8] found that the inferior frontal gyrus (IFG) and inferior parietal lobule (IPL), forming the best studied nodes of the putative mirror neuron system (pMNS), were active when participants engage in different sorts of joint action tasks compared to solo conditions, and have therefore argued that the pMNS could underlie our ability to engage in joint actions [2], [7], [8].

Strictly speaking however, we still know little about the contribution of the pMNS in joint actions as the aforementioned experiments [2], [7], [8] have merely deduced that their activations belong to the pMNS based on the macro-anatomical location of activation. Whether these regions are indeed also active while simply viewing and simply performing motor actions (the definition of the MNS) was not tested in these studies. Given that much of the IFG and IPL does not contain mirror neurons, it is difficult to interpret whether the brain regions in the IFG and IPL identified in these studies are really part of the pMNS, and hence whether the pMNS contributes to joint actions [4], [9].

**Figure 1:**
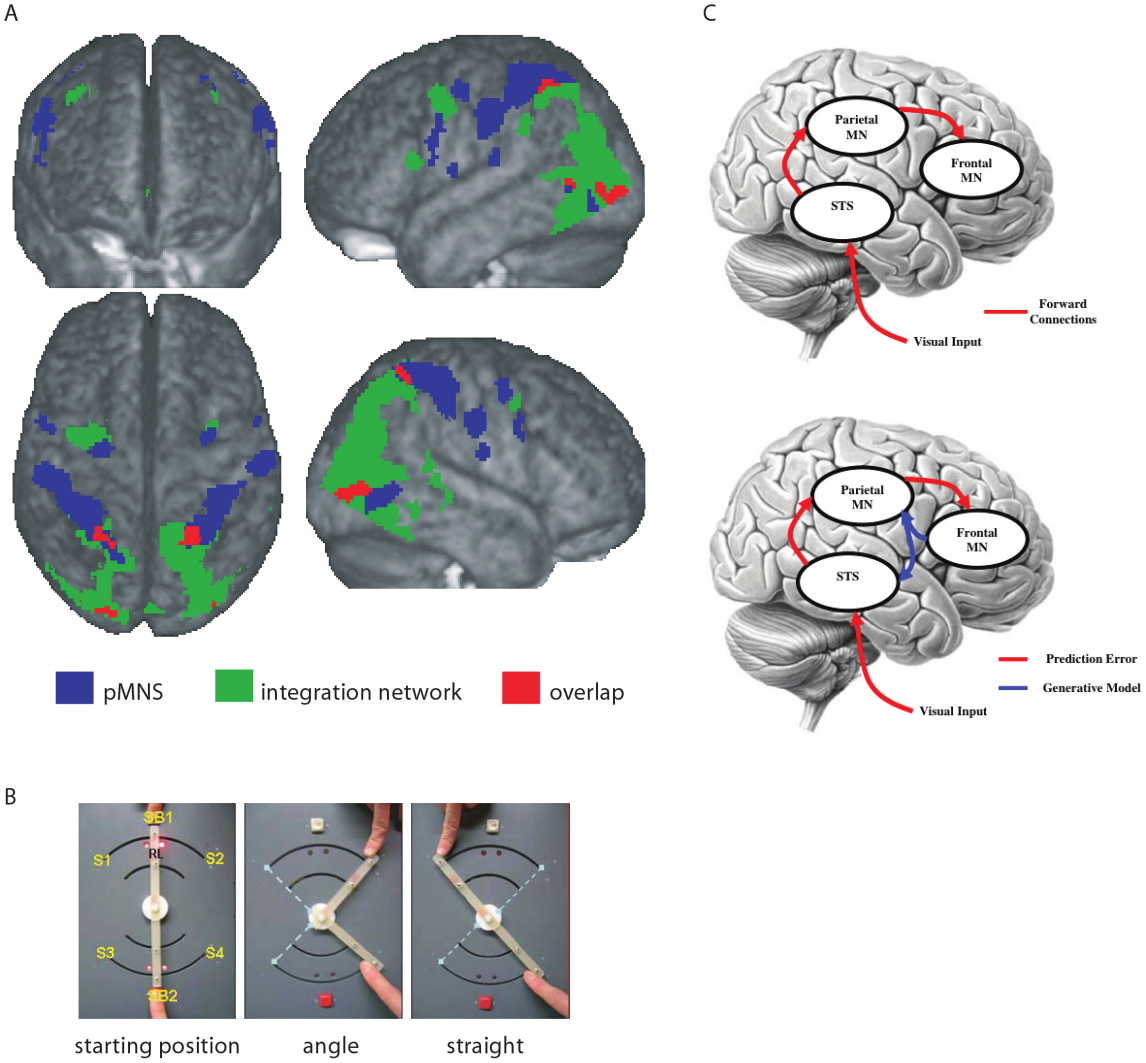
The main results of the fMRI experiment (A), experimental set-up (B) and the schemas of the mirror neuron system (MNS) (C). (A) Rendering of average brain of participants with pMNS (putative mirror neuron system) (blue, exe>0 and obs>0, both p<0.001), integration network (green) and overlap between two networks (red). (B) (left) the photograph of the response box together with the fingers of the experimenter at the top and participant at the bottom; (middle) the correct configuration for an angle trial, dotted lines showing alternative configuration; (right) same for a straight trial. (C) The frontal and parietal sites of the MNS and STS as well as the inverse (recognition) model (red lines) and forward (predictive) model (blue lines) adapted with permission from Kilner JM, Friston KJ, Frith CD (2007) Predictive coding: an account of the mirror neuron system. Cogn Process 8: 159–166.

In order to test the contribution of the pMNS in joint actions more directly, we performed an fMRI experiment in which the pMNS was mapped in addition to mapping regions selectively involved in the integration of the participants' actions with those of the experimenter during joint actions [4]. Participants engaged in joint actions with an experimenter who was standing next to them by creating geometrical shapes in real-time. Participants additionally performed the same actions singly (execution) and observed the experimenter's actions (observation). Consequently, we could identify the common voxels for both execution and observation in order to map the pMNS of our participants. The pMNS was identified in the IFG, precentral gyrus, parietal regions (SI, SII, SPL, see Table S1 for abbreviations) and middle temporal gyrus (MTG) bilaterally (Fig. 1A, blue). We then identified areas where the activity in joint actions exceeded that during the sum of solo execution and observation given that engaging in joint action additionally requires partners to *integrate* solo execution and observation (if integration >0 then joint action  =  observation + execution + integration)> observation + execution). The areas responsible for this *integration* process were located bilaterally in the IFG, precentral gyrus, SPL, IPL, middle and temporal occipital gyri and cerebellum (Fig. 1A, green). Lastly, we checked whether this integration network overlapped with the pMNS to test whether the pMNS directly contributes to joint actions by integrating observed and executed actions. This analysis, however, revealed only very restricted overlap in the SPL and the high-level visual areas (Fig. 1A, red). Indeed, the frontal areas of the integration network in the IFG did not fall in the pMNS. Neither were the anterior sites of the pMNS showing evidence of integrative processes during joint actions. Therefore, this suggested that the anterior sites of the pMNS do not play a *direct* role in the integration of observed and executed actions in joint actions [4]. Instead, we hypothesized that the anterior pMNS sites still contribute to joint actions by transforming observed and executed actions into a single code [10], and then sending this information to regions performing the integration, but not by directly performing the additional integration needed in order to respond with appropriate actions to those of the observed partner [4].

In order to test this hypothesis, here we explore the directional influences between brain areas of the pMNS and the integration network using Granger causality mapping (GCM), which has recently been used to map effective connectivity in the human brain [5], [11], [12], [13]. To maximize the statistical power of this analysis, we did not calculate GCM for the entire brain, but only in the regions that can inform our question: between the anterior sites of the pMNS and the integration network (to examine information flow between the pMNS and integration network) and the posterior sites of the pMNS (to examine information flow within the pMNS) on our fMRI data. All regions were identified in our previous experiment [4].

Firstly, in accord with our hypothesis, our results suggest that these two functionally separate networks were effectively connected. Secondly, this information flow was predominantly backwards (from anterior to posterior regions of the brain) which is compatible with generative models emphasizing that the premotor areas may actually send more predictions to the sensory areas than the other way around. We propose that overcoming the sensory delays by relying on the predicted actions of others could be beneficial when engaging in joint actions which entail the tight temporal coordination of two actors.

## Results

**Figure 2:**
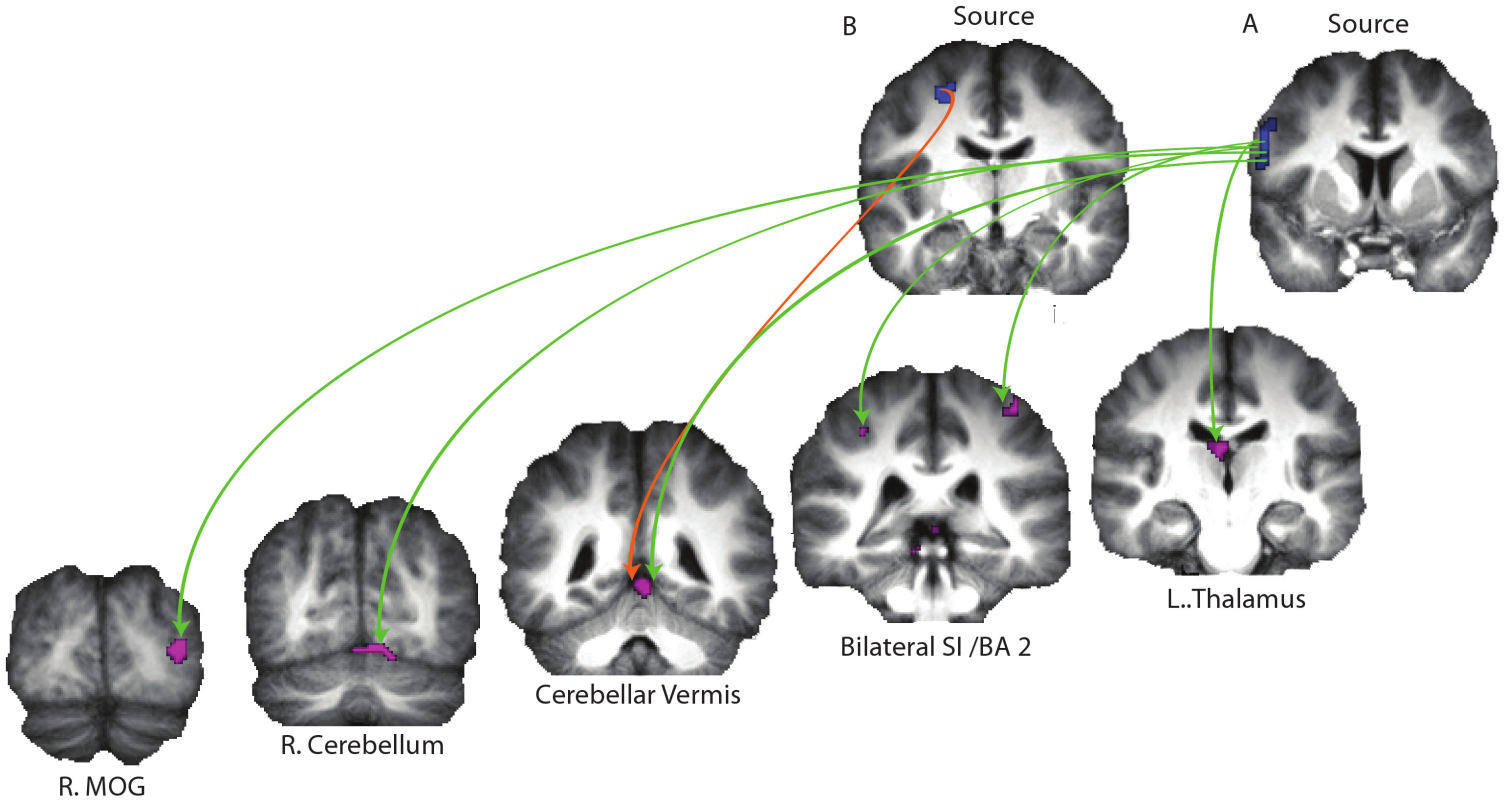
The directed influence of the (A) source region BA44 (blue) and (B) source region BA6 (blue) on the target regions (purple) in the joint action condition. The yellow (A) and orange (B) lines represent the information flow from source regions to the target regions.

The differential Granger Causality (dGC) was significantly above zero from at least some left BA44 voxels (part of the pMNS) to bilateral BA2 voxels in the somatosensory cortex (also within the pMNS) and to voxels of the right MOG, left thalamus, left cerebellar vermis and right cerebellum (within the integration network) when analyses were confined to the joint action blocks (Fig. 2A & Table 1). This suggests that some voxels in the left BA44 sent significantly more information to some voxels of both the pMNS and the integration network than it received from voxels in these regions during joint actions. In addition, the dGC was significantly above zero from at least some voxels in left BA6 (part of the pMNS) to voxels of the left cerebellar vermis (part of the integration network) when analyses were confined to the joint action blocks (Fig. 2B & Table 2). This suggests that some voxels in the left BA6 influenced the left cerebellar vermis part of the integration network more than the other way around during joint actions.

**Table 1:** The directed influence of the source ROI 1 (BA 44) on the target regions in the joint action condition revealed with GCM.

Source	Target	Network*	Hem	x	y	z	size(vx)
BA 44 (ROI 1)	SI/BA2	pMNS	L	−36	−35	48	10
	SI/BA2	pMNS	R	40	−33	63	13
	c. vermis	int. network	L	−3	−39	−6	33
	cerebellum	int. network	R	21	−66	−15	30
	thalamus	int. network	L	−6	−18	18	18
	MOG	int. network	R	39	−84	12	30

*The network that the target is part of. Two networks were identified in a previous study [1]: the putative mirror neuron system (pMNS) and integration network (int. network).

**Table 2:** The directed influence of the source ROI 2 (BA 6) on the target regions in the joint action condition revealed with GCM.

Source	Target	Network	Hem	x	y	z	size(vx)
BA 6 (ROI 2)	c. vermis	int.network	L	−3	−39	−6	26

**Figure 3:**
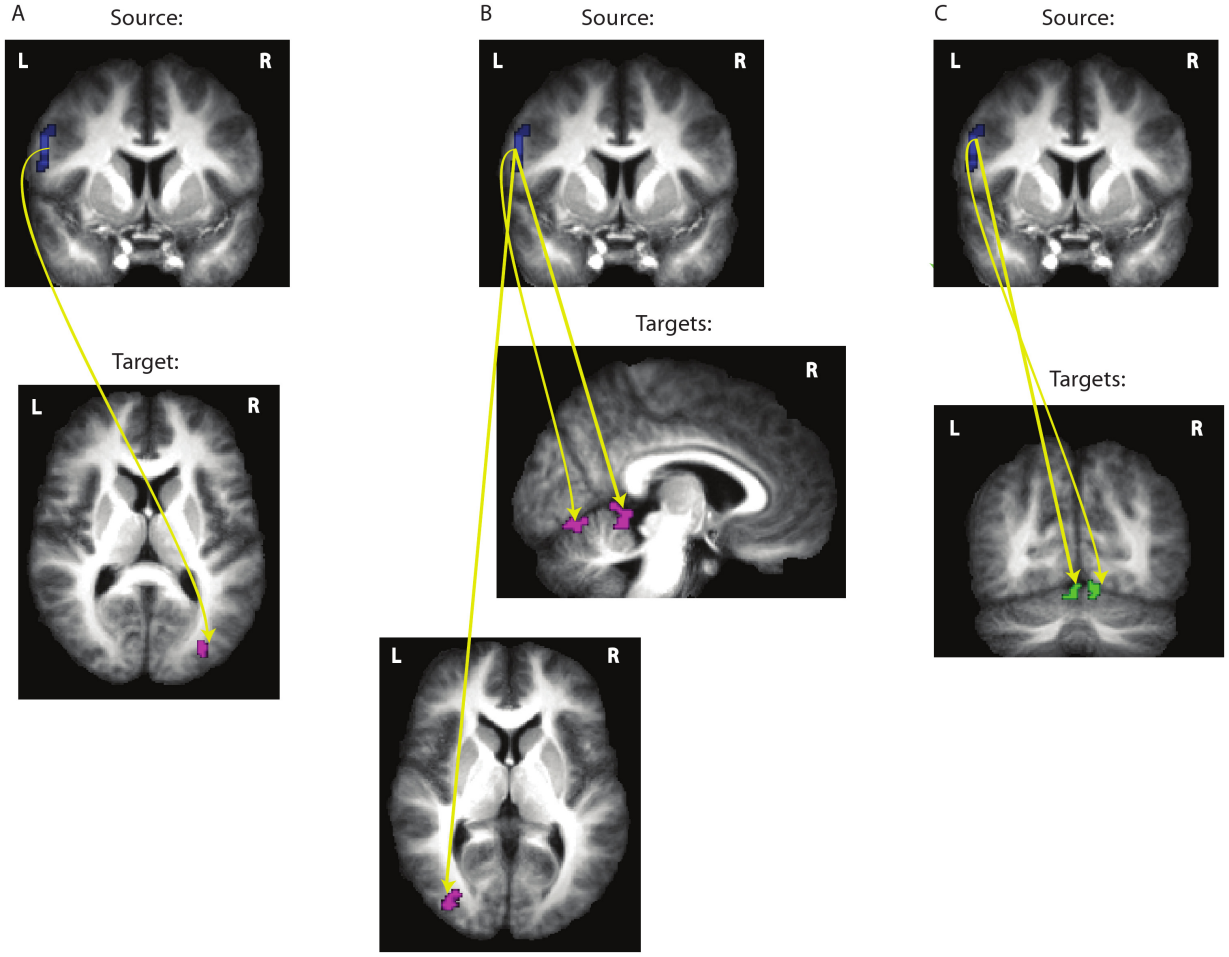
The directed influence of the source region BA44 (blue) on the target regions (purple) in the execution (A) and observation (B) conditions. (**C**) The difference in directed influence of the source regions BA44 (blue) on the target regions (green) between the joint action and execution conditions (joint action>execution). The yellow lines represent the information flow from source regions to the target regions.

On the other hand, when analyses were confined to the solo execution blocks, we found that the dGC was significantly above zero only from some left BA44 to some right MOG voxels (Fig. 3A & Table 3). This suggests that during solo execution in which the participants moved their stick to left or right, parts of the left BA44 sent significantly more information to parts of the right MOG, a high level visual area within the integration network, than it received. When analyses were confined to the observation blocks, we found that the dGC was significantly above zero only from some voxels of the left BA44 to some voxels in the bilateral cerebellar vermi and left MOG (Fig. 3B & Table 4). Thus, during solo observation in which the participants observed the experimenter moving her stick to left and right, the left BA44 sent significantly more information to two regions of the integration network in bilateral cerebellar vermi and left MOG than it received from them. We did not find any significant dGC above zero between the anterior and posterior sites of the pMNS when analyses were confined to the observation and execution blocks.

**Table 3:** The directed influence of the source ROI 1 (BA 44) on the target regions in the execution condition revealed with GCM.

Source	Target	Network	Hem	x	y	z	size(vx)
BA 44 (ROI 1)	MOG	int. network	R	36	−81	9	17

**Table 4:** The directed influence of the source ROI 1 (BA 44) on the target regions in the observation condition revealed with GCM.

Source	Target	Network	Hem	x	y	z	size(vx)
BA 44 (ROI 1)	c. vermis	int. network	R	3	−45	−3	33
	c. vermis	int. network	R	3	−72	−9	33
	MOG	int. network	L	−36	−87	3	23

In addition, a direct comparison between differential Granger Causality maps (dGCMs) calculated for the various conditions (i.e. joint action versus execution blocks and joint action versus observation blocks) revealed significant differences in the dGCMs originating from some left BA44 voxels to voxels of the bilateral cerebellum: values were significantly larger during joint action blocks compared to execution blocks (Fig. 3C & Table 5). This suggests that the directed influence of left BA44 on bilateral cerebellum was significantly stronger during joint actions than during execution. Similar direct comparison of dGCMs between joint action and observation blocks did not reveal any significant difference.

**Table 5:** The difference in directed influence of the source ROI 1 (BA 44) on the target regions between the joint action and execution conditions (joint action>execution) revealed with GCM.

Source	Target	Network	Hem	x	y	z	size(vx)
BA 44 (ROI 1)	cerebellum	int. network	R	9	−69	−18	19
	cerebellum	int. network	L	−3	−69	−15	13

## Discussion

In our previous fMRI study, in contrast with other studies and theoretical accounts suggesting a central role for the pMNS for action integration in joint actions [2], [7], [8], we previously showed that the brain areas responsible for integration during joint actions fall outside the pMNS, especially in the anterior sites of the pMNS (the inferior frontal gyrus (BA44) and precentral gyrus (BA6)) [4].

In the present study, we further explored the contribution of the pMNS in joint actions with a different method: GCM. We tested our hypothesis, formulated previously, that the anterior sites of the pMNS may not participate in integration directly, but do so indirectly by transforming observed and executed actions into a common code [4], [10], [14] and feeding this information to other brain regions that can then integrate the commonly coded action representations depending on the goal of a particular trial [4]. More specifically, we thought that Hebbian associations occur in the pMNS between performing a certain action and seeing and feeling that action [15], [16]: While the participant saw himself perform certain actions in the past, Hebbian learning would have strengthened synaptic connections between neurons in the visual cortex that respond to the sight of his action, neurons in BA2 that represent proprioceptive information from the moving limb, and neurons in BA6 and 44 that triggered the action. Because of the bidirectional nature of the connections between these regions [16], such connections would be strengthened both in the motor to visual and somatosensory and in the visual and somatosensory to motor direction. During action observation, seeing someone else perform a similar action would then trigger activity in BA2, BA6 and BA44 neurons involved in performing a similar action due to the visual similarity with our own movement triggering, through these strengthened synaptic, those premotor and somatosensory neurons which activity was associated with that of the visual neuron in past performances of the same action. At the same time, during motor execution, these strengthened synaptic connections would trigger activity in BA2 and visual neurons representing the visual and proprioceptive consequences of our own movements.

Simple reaction time experiments [17], [18] shows that it takes humans between 200 and 300 ms to process the simplest visual stimuli and generate a simple motor response to that stimulus. Because, when we perform an action, it therefore also takes 200–300 ms for the chain of event that could cause Hebbian learning (motor activity in BA6/44 → overt movement of the arm → activity in the retina and peripheral somatosensors → activity in the visual and somatosensory cortices → synaptic input back to BA6/44) and these Hebbian associations will develop predictive properties. For instance, while reaching to slide an object aside, by the time the visual cortex is representing the reaching movement and BA2 responds to the proprioceptive feelings of the arm reaching, the premotor cortices (BA6 and 44) will already trigger the sliding phase of the movement that follows reaching after 200–300 ms. Accordingly, Hebbian associations will strengthen synapses between seeing (visual cortex) and feeling (BA2) a movement and programming the next movement (BA6 and 44) that normally follows the first movement after 200–300 ms. While seeing the actions of others, our brain would still take 200–300 ms to respond to its perception of the actions of others (as in any reaction time task) but the neural representation of the actions of others in BA6/44 would be a representation of the actions most likely to occur in 200–300 ms. The motor reaction time and predictive horizon would then precisely cancel each other out, and the brain could actually act in synchrony with others instead of lagging behind.

Through this set of associations, seeing the actions of others will trigger expected somatosensory and motor representations, and vice versa, programming ones own actions would trigger visual and somatosensory representations. Viewing the actions of others and performing ones own actions would therefore be associated with the same type of representations in motor, somatosensory and visual representations. Brain regions that represent a particular goal to be achieved with a partner (i.e. creating an angle or straight line in our experiment) need to coordinate the participant's own actions with what he sees another individual perform. This task would become computationally more difficult, if the participants' own actions were represented in a different code than those of the observed agent, and simpler, if they were represented in the same code. The pMNS could therefore facilitate the role of such integrative brain regions by doing what it seems to do: representing the participant's and the experimenter's (anticipated) actions in the same code [10], [19].

Here we show that during joint actions, anterior sites of the pMNS in the left hemisphere (BA44 and dorsal BA6) indeed were exchanging information with the integration network during joint actions: GCM showed that left BA44 sent significantly more information to than it received from the integration network (right MOG, left thalamus, left cerebellar vermis and right cerebellum). In addition, left BA6 exchanged information with the cerebral vermis by sending significantly more information than receiving from it. Thus, the anterior sites of the pMNS could play a role in the integration of actions during joint actions by feeding information into the areas that are part of the integration network suggesting that these two anatomically separate networks (the pMNS and integration network) work in concert during joint actions.

In addition, our analysis showed that during joint actions the anterior sites of the pMNS also sent information to BA2 within SI which was part of our pMNS network, because it was active both during the solo observation and execution of the actions in our task [4]. Although single cell recordings in BA2 have so far not systematically explored the presence of mirror neurons, reviews of the literature [20], [21] provide strong evidence for the fact that BA2 is systematically activated while we perform and observe the actions of others. This suggests that the pMNS is constituted of two branches: the classic motor branch and a less explored somatosensory branch that represents the proprioceptive and tactile input one would experience when performing similar actions [21]. The information flow we found here between BA44 and BA2 therefore suggests that these two branches interact during joint actions, with the motor branch possibly triggering representations of the expected somatosensory consequences of the observed and/or planned actions.

Apart from providing empirical evidence for information exchange within the pMNS and between the pMNS and integration network in the service of joint actions, our results also revealed the predominant direction of the underlying effective connections: the information flow was predominantly backwards (i.e. from frontal left BA44 to more posterior areas: bilateral BA2, right MOG). Recent simulations performed by Schippers and colleagues (manuscript in preparation) suggest that even in the presence of variability in hemodynamic response latency between different brain regions, the detected direction of prevalent information flow detected using differential GCM within a condition correctly identifies the true direction of information flow in >80% of the cases. Another study using simulations demonstrated that GC could be used to infer neuronal causality reliably (accuracies up to 90%) in the presence of neuronal delays [22]. We will, therefore, discuss how the backwards information flow detected in our study is compatible with the increasingly prominent concept of generative/forward models [14], [16], [23], [24], [25], [26], [27], [28].

It has been proposed that while the forward connections from visual to premotor regions form inverse models through which the visual information is converted to predicted motor plan; the backwards connections, from premotor to visual and somatosensory regions generate the predicted sensory outcome of the action representations triggered in premotor regions, forming forward (generative) models [12], [14], [16], [23], [24], [25], [26], [27], [28], [29], [30]. In this concept, the premotor cortex is part of both forward and inverse models (Fig. 1C) and closes a loop of information flow circling between premotor, somatosensory and visual areas which could play a key role in social actions [14], [23], [25], [31], [32], particularly in the case of joint actions requiring tight temporal synchrony between cooperative partners. As mentioned above, based on simple reaction time experiments, it takes us about 200–300 ms to respond to a stimulus. Accordingly, instead of being synchronized with the actions of others, our actions would lag several hundreds of milliseconds behind the perceived actions of our partner. However, humans can do much better than that. In the well-studied case of music [33], it has become apparent that two musicians can synchronize their performance to each other's timing with asynchronies of ∼30 ms [34], which is also the threshold at which humans typically perceive two notes as asynchronous [35]. Although an example of auditory-motor rather than visio-motor synchrony, joint music playing shows that the brain can overcome sensory-motor delays to reduce inter-individual asynchronies below the perceptual threshold. The fact that people actually tap slightly ahead of a beat they should synchronize with [36], [37], [38], [39], suggests that predictions probably play a role, and the fact that musicians are better at synchronizing with prerecorded pieces they played themselves supports the idea that motor simulation, as described in our Hebbian learning scenario above, could play a role [34]. Accordingly, it has been proposed that one way to be able to synchronize one's actions with others or external stimuli would be to base our motor planning not on actual (and therefore delayed) sensory input, but on the prediction of forthcoming actions, as provided by generative/forward models [4], [14], [23], [25], [26], [27], [40]. However, so far, empirical evidence for such generative models in joint action experiments remains scarce.

In our experimental design, each trial was composed of an unpredictable beginning and a more predictable continuation. At the commencement of each trial, the participant needed to detect the side the experimenter decided to move her part of the device towards. This phase was relatively short (<500 ms). Thereafter, the participant could predict how the movement of the experimenter would continue in the remaining 1.5 s, and s/he only needed to adjust her/his own actions to this predictable trajectory. In this context, generative models predict the predominant direction of information flow that should occur in the brain. Only in the initial 0.5 s should information flow predominate in the ‘forward’, visual to premotor direction because predictions cannot yet be formed accurately. During the longer remaining time (∼1.5 s), predictions can be formed, and information flow in the ‘backwards’, frontal to visual and somatosensory direction should build up. Given that these ‘backward’ flowing predictions are known to cancel the neural representation of expected visual information from the visual cortex [41], [42] and expected somatosensory information from the somatosensory cortex [43], the forward information flow in the visual to frontal and somatosensory to frontal direction should be much reduced. Overall, integrating these predictions over the entire duration of the trial, information flow in the backwards, premotor to visual and somatosensory direction should therefore prevail. The fact that this is exactly what we measured in our experiment provides support for the notion that the pMNS is part of forward/generative neural model [14], [16], [23], [25] that could play a key role in joint actions not only by transforming observed and executed actions in a common code but also by computing predictions that can overcome otherwise inevitable neural delays.

In addition to the predominantly ‘backwards’ cortical information flow we also found that both left BA44 and BA6 sent more information to the cerebellum and cerebellar vermis, than they received from it, respectively. These regions of the cerebellum play important roles in motor control and are thought to be part of the forward models central to every form of skilled motor control [27], [44], [45], [46], [47], [48]. During motor control, the convergence of input from the premotor cortex and sensory structures makes the cerebellum an ideal cite for calculating in real time the error between intended and actual movement, and using this error to improve motor performance [49]. These real time calculations seem to be important for the actions performed by individuals in solo conditions [40]. One might speculate, that during joint actions, the cerebellum may play a similar integrative role in detecting errors in synchrony between ones own actions and those of others. The fact that the cerebellum receives more input from the premotor cortex during joint actions compared to solo motor execution suggests that during joint actions, the cerebellum might receive information about the (predicted) actions of the other agent in addition to the intended motor command that the cerebellum is known to receive from premotor cortices during solo action execution. This would provide the cerebellum with the information it would need to fine tune the way in which the actions of the two agents need to be coordinated to achieve their common goal.

At first sight, one might however wonder why we failed to find any brain region that primarily sends information to the frontal pMNS regions. After all, these regions have to receive visual information about the actions of the partner from somewhere. It is important to keep in mind however that GCM analyses of fMRI data employ a *differential* Granger approach [5]. Simulations have shown that GCM applied to fMRI signals cannot accurately infer whether information is sent from one region to another per se, however it can establish whether *more* information is sent from one region to another than vice versa [5]. By following that approach, our analyses showed that during joint actions, information flow is *more* pronounced in the premotor to somatosensory direction than vice versa. Our analyses therefore do not show that the frontal pMNS regions do not receive information from more posterior regions, nor that there is no information flow between regions where our analysis find no significant differential GC - however that more information is sent in the backwards direction, as expected by generative models. Testing the concept of a loop of information would need data of higher temporal resolution to separate the first couple of hundreds of ms from the rest of the trial. Thus, in the future, we plan to use methods with higher temporal resolution (EEG or MEG) in order to investigate interactions at a time scale closer to that of the neural processing itself and to explore the prediction that the predominant direction of influence shifts between the unpredictable beginning and the predictable continuation of each trial.

It had been suggested that because various brain regions can differ in their hemodynamic response function, and Granger causality is based on temporal precedence, differential Granger causality (dGC) might indicate information flows from A→B simply because region A has a faster hemodynamic response than region B [5]. To avoid such biases, Roebroeck and colleagues suggested to look at dGC in different conditions, and to focus on dGC results that are present in one but absent in another condition [5]. Directly contrasting dGC between two conditions was not performed in their paper. Using such a conservative approach instead of interpreting all significant dGC results obtained from our joint action blocks, the main message of our paper remains unchanged. The anterior pMNS regions evidenced significant positive dGC values with BA2 during the joint action condition but not the solo conditions, providing further evidence for information flow in the anterior to posterior direction within the pMNS during joint actions. There was also an increased dGC from anterior pMNS to the cerebellum during joint actions compared to solo motor execution, providing further evidence for the interaction between the pMNS and the integration network in our task. This suggests that the information transfer within the pMNS and between the pMNS and the integration network is indeed specific for cases in which participants need to coordinate their own actions to external stimuli, such as was the case during joint actions. Our own data simulations, performed by Schippers and colleagues (manuscript in preparation), using realistic differences in hemodynamic response functions and a group analysis approach, however suggest that the direction of predominant information flow derived from a single condition is accurate in over 80% of the cases suggesting that the results of the analysis in the joint action condition alone can be interpreted more safely than Roebroeck and colleagues had suggested [5].

In summary, we present evidence that there is information flow from the anterior sites of the pMNS to the integration network, i.e. the bilateral cerebellum and maybe (if single condition dGC can indeed be interpreted safely as suggested by Deshpande et al. (2010) and Schippers et al., in preparation) right MOG, left thalamus and left cerebellar vermis, and posterior sites of the pMNS, i.e. BA2. This sheds new light onto the role the pMNS during joint actions. As suggested by our previously published traditional analysis of this data, the pMNS does not seem to be directly involved in the task dependent integration of observed and executed actions during joint actions [4]. However, the pMNS could contribute to joint actions indirectly, by transforming observed and executed actions into a common code and being part of a generative model that could predict the future somatosensory and visual consequences of observed and executed actions in order to overcome neuronal delays. This information is then sent to regions such as the cerebellum that can integrate our own actions with those of others and permit the exquisite temporal coordination characterizing so many joint actions.

## Methods

We employed Granger causality mapping on fMRI data collected for a previous experiment [4]. Temporal information in the data was used to measure the influences between brain regions without an apriority model of regional connections. The procedure of the fMRI experiment was published previously [4]. Here we report the relevant details for this study only.

### Ethics Statement

The experiment was approved by the Medical Ethical Commission of the University Medical Center Groningen, the Netherlands. Participants gave informed consent and were paid for their participation.

### Participants

18 healthy volunteers; all right-handed; 10 female and 8 male; mean age 23.7 years ranging 20–45 years with normal or corrected to normal vision and without a history of neurological, major medical, or psychiatric disorders. The experiment was approved by the Medical Ethical Commission of the University Medical Center Groningen, the Netherlands. Participants gave informed consent and were paid for their participation.

### Procedure

In the fMRI session, the participant played a cooperation game with the experimenter who was standing next to the participant (joint actions) or performed one of two non-cooperative control conditions (solo execution and solo observation).

#### 1) Joint Action

The experimenter and the participant together shaped the two sticks of a game box in either an ‘angle’ or a ‘straight’ line (see Fig. 1B and Video S1). Each stick was controlled by a different player: the lower stick was controlled by the participant and the upper one by the experimenter.

In the beginning of each trial, players had to press their respective start buttons and hold their right index fingers there (SB1 and SB2 in Fig. 1B) until the experimenter started to move her stick. In the meantime, each of them received different auditory instructions: the experimenter received auditory instructions indicating where (left or right) she should move her stick; the participant received auditory instructions indicating the geometrical shape (an angle or a straight line) that they would need to create together. Between 1 and 2s (random interval) after the participant had received the angle or straight instruction the experimenter was instructed with a ‘go’ signal. Immediately after this signal, she initiated the joint action by moving the upper stick to the left or right while the participant had to react by starting to slowly move the lower stick in the direction suitable to achieve the target shape (Video S1). Likewise, the participant had to synchronize his actions closely with those of the experimenter to reach the target location virtually simultaneously (within 200 ms of each other) to jointly win the trial. This tight time constraint ensured that both players monitored and coordinated the velocity of their movements carefully and continuously throughout the trial, requiring both the spatial and temporal coordination that defines joint actions. Consequently, participants had to carefully watch the experimenter's actions to determine (a) *which side* to move and (b) *when* and how quickly to move.

#### 2) Execution (exe)

In the joint action condition, a red light (RL in Fig. 1B) was turned on whenever the experimenter placed her finger on the start button (SB in Fig. 1B) and turned off whenever she left the SB to start her action. Likewise, in the execution condition, the experimenter's RL was turned on and off with the same timing as in the joint action conditions without the experimenter being visible. The participants were instructed to move their stick to the right or left whenever they saw the red light turn off on the box, ensuring that the timing of the participant's actions was the same as in the joint action blocks but not triggered by a biological action. The participants could choose what side to go to, but were instructed by the experimenter to avoid going to the same side constantly. At the end of both joint action and execution trials, they released their sticks and had to place their index finger back onto the starting buttons and wait for the auditory instructions of the next trial.

#### 3) Observation (obs)

Participants were instructed to carefully watch the experimenter move her stick randomly to the right or left using the same timing as in a joint action block.

Different conditions were arranged in blocks of 8 trials separated by 2.3 s. Each trial lasted between 3.6 and 4.6s depending on the random interval between the auditory trial instruction and the initiation of the movement. Accordingly, each block lasted between 45 and 54s depending on these random intervals. Blocks were separated by 14±2 s random pauses (including the verbal instruction or the sound indicating the type of block to follow). Each block started with a different auditory instruction presented 1.75 s before the block indicated the nature of the block: for the execution block ‘action’ (400 ms), for the observation block ‘look’ (400 ms) words were presented; for the joint action blocks a sine wave (440 Hz) tone was presented. The experiment also contained a sound only condition which was however not used in this GCM analysis.

Each run contained two blocks of each of the conditions and five runs (a total of 10 blocks of each condition) were acquired. The order of the conditions was counterbalanced between runs and participants. Stimuli were programmed and presented using the Presentation software (Neurobehavioral systems, Davis, CA). At the end of the each run, participants were informed about how successful they were in creating shapes in the joint action trials to convey a mutual feeling of cooperation.

### Data acquisition

Imaging was performed with a Philips Intera 3T Quaser with a synergy SENSE head coil and maximum gradient strength of 30 mT/m. Head movements were minimized by using foam padding and never exceeded 3 mm in a run. We used a standard single shot EPI with TE  = 28 ms, TA = 1.25 s, TR = 1.3 s, 28 axial slices of 4 mm thickness, without slice gap and a 3.5×3.5 mm in plane resolution acquired to cover the cortex and most of the cerebellum. A T1 weighted structural scan was acquired with TR = 15.31 ms, TE = 3.6 ms, flip angle = 8 deg.

### Data preprocessing

Using SPM5 (www.fil.ion.ucl.ac.uk/spm) implemented in MATLAB 6.5 (Mathworks Inc., Sherborn, MA, USA). All EPI volumes were aligned to the first volume acquired for each participant and a mean EPI image generated after realignment. Spatial normalization was performed by co-registering the structural volume to the mean EPI, segmenting the coregistered structural image, determining the normalization parameters required to warp the gray matter segment onto the gray matter MNI template, and applying these parameters to all EPI and structural volumes. No spatial smoothing was applied on the functional data for the granger data, but smoothing had been used to calculate the traditional GLM published previously [4] which served to delineate our regions of interest for the Granger analysis.

### Traditional GLM analysis at the Single participant level

GLM was performed using separate auditory predictors for the conditions joint action condition to capture brain activity caused by hearing the words “angle” or “straight” and separate action predictor for the joint action, observation and execution conditions to capture brain activity triggered by executing and/or observing the finger movements. Each predictor was a boxcar function that reflected the trial-by-trial timing of the auditory and movement epoch of the condition. The boxcar functions were convolved with the haemodynamic response function, and fitted separately for each run to the data. In addition, the head motion and rotation along the three axes were entered as six covariates of no interest in the design matrix to single out motion artifacts although motion never exceeded 3 mm within a run. Given that little time separated the auditory instructions from the actions within a block (average = 1500 ms), the auditory and action predictors overlap in time (after convolution with the haemodynamic response function), and the attribution of a brain activity to one rather than the other was uncertain. Instead of analyzing the parameter estimates separately for the auditory and action predictor, we combined them by summing the surface under the fitted auditory and action predictors. This was done simply by multiplying the parameter estimates (Beta) obtained from the GLM with the surface (S) under their respective predictor (*S = Beta_auditory_xS_auditory_ + Beta_action_xS_action_*). Brain activity across conditions were compared using this surface (for details please see [4]).

### Population analyses

To implement a random effect analysis, contrast estimates obtained separately for each participant were tested at the population level, using one-sample t-tests and ANOVA analyses testing whether the average contrast differs from zero. Only results that are significant both at p<0.001 uncorrected and p<0.05 corrected using false discovery rate are reported as significant. Only clusters of at least 10 voxels are shown.

### pMNS (Putative Mirror Neuron System) definition

First, we compared the surface under the curve in *obs* against zero (t-test) and we did the same for *exe*, too. Later, only those voxels with significant results (p<0.001, uncorrected) in both analyses at the second level were identified and constituted the pMNS (i.e. (Beta_action_xS_action_)_obs_>0 & (Beta_action_xS_action_)_exe_>0, where & is a logical, both at p_unc_<0.001 and p_fdr_<0.05).

### Integration Network definition

To map the regions showing activity that indicates their contribution in integrating observed and executed actions, two contrasts were calculated for angle and straight joint actions by subtracting both *obs* and *exe* from joint actions with the surface analysis at the first level. Later 18 contrasts (one per participant) per joint action (*ang* & *str*) were entered in a one way ANOVA without constant, and the global null conjunction calculated to estimate the likelihood of the null hypothesis (m(*C_ang_*)< = 0 & m(*C_str_*)< = 0) that the voxel was not involved in either joint action [50]. To prevent to accept the voxels in which the activity in angle (*ang*) or straight (*str*) was above the sum of execution and observation (*exe+obs)* without being above the activity in *exe* and *obs* individually, we required that these voxels fall within an inclusive mask where ((*ang*>*exe* and *ang*>*obs*) or (*str*>*exe* and *str*>*obs*)).

### Single Subject Granger Causality Mapping (GCM)

GCM is an effective connectivity method, which is based on the Granger causality concept to measure the existence and predominant direction of influence from information in time series [5]. The concept of Granger causality states that if a time series I_t_ has a causal influence on J_t_, then fluctuations in I_t_ should consistently precede those in J_t_. More specifically, I_t_ is thought to Granger cause J_t_ if taking I_t-1_ into account improves the capacity to predict J_t_ compared to only taking J_t-1_ into account. Here, differential Granger causality maps (dGCM) were computed for each source voxel with an order of 1 TR (1.3s) by computing the linear direct influence of source to target (I→J) and of target to source (J→I), and subtracting the latter from the former. This was done separately for the three block/conditions (joint action, execution and observation) using unsmoothed normalized data. All calculations were performed using an in-house program coded in MATLAB (The Mathworks, Natick, MA) which uses SPM5. The input to the program consisted of the time course of each source voxel (I) which was extracted using MarsBar (http://marsbar.sourceforge.net) and a binary temporal mask (M(t)) which had a value of 1 if this volume fell within an epoch to be included in that particular calculation and a 0 otherwise. Our in-house software then only used those volumes of the data for M(t) and M(t-1) has a value of 1 to calculate directed influences with an order of 1. The influence of region X on region Y was quantified by calculating two regressive models taking all t into account for which M(t) = M(t-1) = 1. One predicting the present of variable Y only based on the past of Y itself: Y(t) = a*Y(t-1)+e(t); and one additionally taking the past of X into account Y(t) = a'*Y(t-1)+b*X(t-1)+e' (t). The influence of X→Y is then calculated as F_X→Y_ = sd(e(t))/sd(e' (t)). This means that we essentially concatenated all repetitions of each type of block (total of 10 repetitions per condition in the experiment) and we used these concatenated time series to calculate the single autoregressive model but avoiding the borders between repetitions of the blocks where the Y(t) would be explained by values taken from a previous block acquired more than 1.3s ago. As recommended [5], both the temporal mask was restricted to the steady state phase of each block (i.e. the volumes corresponding to 10 s after the block onset), and were calculated separately for the joint action condition, the solo execution and the solo observation condition.

### Source Regions of Interests for the GCM Analysis

Based on our hypothesis, the anterior sites of the pMNS (in BA44 and in BA6) in the left hemisphere were selected as source region of interests (ROIs) based on their task-dependent BOLD characteristics as identified as significant clusters in the random effects group analysis in our previously published analysis [4]. The labels for the clusters are based on the cytoarchitectonic areas (based on the anatomy toolbox [51] for SPM). The details of the ROIs can be found in Table 6.

**Table 6:** Source Regions of Interests for the GCM.

Source ROI	Area (Anatomy)	Area (BA)	Network	Hem	x	y	z	size(vx)
ROI 1	IFG	BA 44	pMNS	L	−58	8	26	26
ROI 2	preCG	BA 6	pMNS	L	−26	−10	52	22

### Second level differential GCM analysis

To directly test whether any voxels in the anterior left regions of the pMNS exchange information with other regions of the pMNS or integration network, we then performed the following second level analysis. We applied smoothing with a Gaussian kernel of 8 mm full-width at half maximum (FWHM) to all dGCM maps of all participants (which have been calculated using unsmoothed data) to account for differences in localization across participants. These were 26 dGCM for the BA44 ROI and 22 dGCM for the BA6 ROI for each participant, corresponding to each of the voxels in the two ROIs. The smoothed 26 dGCMs for all the voxels of the BA44 ROI were then included in a one-way ANOVA with dependent variance in order to account for spatial autocorrelation between neighboring voxels. We then defined a separate t-contrast for each of the dGCM (corresponding to each of the n voxels within BA44), and performed a global null conjunction including all these t-tests using SPM and the minimum t-statistics [50]. This tests the null hypothesis that none of the voxels in BA44 has a dGCM larger than zero. We used this procedure instead of simply using the average time course of the entire ROI to detect cases in which only some voxels within our ROI have significant non-zero dGC while controlling for the multiple comparison problem arising from testing n contrasts (for further details see [50]). Additionally, by explicitly masking this conjunction with a mask including all other regions of the pMNS and integration network, we directly tested whether BA44 sends more information to than it receives from all other voxels in the pMNS or integration network. The same was performed using t-test examining if each dGCM was significantly smaller than zero to test if any voxel received more information from than it sends to any of the other regions of the pMNS and integration network. The same procedure was then performed for the BA6 ROI, too. In addition, we calculated a direct comparison between differential Granger causality maps between the various conditions (i.e. joint action versus execution blocks and joint action versus observation blocks). All the procedure which was used for within condition calculation was same (as described above for an example ROI, BA44,) except the following: by using ImCalc function of SPM we applied a subtraction between GCMs of different conditions (i.e. GCMs _joint action_ - GCMs _execution_) for each voxel for all ROIs. The resulting difference GCMs then entered in one-way ANOVA. We threshold our results at p<0.001 at the voxel level and corrected for multiple comparisons at p<0.05 with false discovery rate (FDR) and used a minimum cluster size of 10 voxels. We then overlaid our results onto an average brain of our participants for displays. All results were threshold using FDR correction at p<0.05.

## Supporting Information

Table S1 Abbreviations used in the paper together with their meanings.
(0.05 MB DOC)

Video S1 An *angle* joint action trial. This video illustrates an example of an ang condition where the participant and the experimenter cooperates to create an angle shape. The experimenter's hand can be seen on the upper part of the response box, whereas the participant's is on the lower part.
(0.51 MB MOV)

## References

[1] Newman-Norlund RD, Noordzij ML, Meulenbroek RG, Bekkering H (2007). Exploring the brain basis of joint action: co-ordination of actions, goals and intentions.. Soc Neurosci.

[2] Newman-Norlund RD, van Schie HT, van Zuijlen AM, Bekkering H (2007). The mirror neuron system is more active during complementary compared with imitative action.. Nat Neurosci.

[3] Knoblich G, Jordan S, Gallese MISV (2002). The mirror system and joint action.. Mirror Neurons and the Evolution of Brain and Language.

[4] Kokal I, Gazzola V, Keysers C (2009). Acting together in and beyond the mirror neuron system.. Neuroimage.

[5] Roebroeck A, Formisano E, Goebel R (2005). Mapping directed influence over the brain using Granger causality and fMRI.. Neuroimage.

[6] Sebanz N, Bekkering H, Knoblich G (2006). Joint action: bodies and minds moving together.. Trends Cogn Sci.

[7] Newman-Norlund RD, Bosga J, Meulenbroek RG, Bekkering H (2008). Anatomical substrates of cooperative joint-action in a continuous motor task: virtual lifting and balancing.. Neuroimage.

[8] Sebanz N, Knoblich G, Prinz W, Wascher E (2006). Twin peaks: an ERP study of action planning and control in co-acting individuals.. J Cogn Neurosci.

[9] Thioux M, Gazzola V, Keysers C (2008). Action understanding: how, what and why.. Curr Biol.

[10] Etzel JA, Gazzola V, Keysers C (2008). Testing simulation theory with cross-modal multivariate classification of fMRI data.. PLoS One.

[11] Goebel R, Roebroeck A, Kim DS, Formisano E (2003). Investigating directed cortical interactions in time-resolved fMRI data using vector autoregressive modeling and Granger causality mapping.. Magn Reson Imaging.

[12] Jabbi M, Keysers C (2008). Inferior frontal gyrus activity triggers anterior insula response to emotional facial expressions.. Emotion.

[13] Schippers MB, Roebroeck A, Renken R, Nanetti L, Keysers C (2010). Mapping the information flow from one brain to another during gestural communication.. Proc Natl Acad Sci U S A.

[14] Gazzola V, Keysers C (2009). The observation and execution of actions share motor and somatosensory voxels in all tested subjects: single-subject analyses of unsmoothed fMRI data.. Cereb Cortex.

[15] Del Giudice M, Manera V, Keysers C (2009). Programmed to learn? The ontogeny of mirror neurons.. Developmental Science.

[16] Keysers C, Perrett DI (2004). Demystifying social cognition: a Hebbian perspective.. Trends Cogn Sci.

[17] Adam JJ, Van Veggel LM (1991). Discrete finger response latencies in a simple reaction time task.. Percept Mot Skills.

[18] Michie PT, Clarke AM, Sinden JD, Glue LC (1976). Reaction time and spinal excitability in a simple reaction time task.. Physiol Behav.

[19] Keysers C, Kohler E, Umilta MA, Nanetti L, Fogassi L (2003). Audiovisual mirror neurons and action recognition.. Exp Brain Res.

[20] Caspers S, Zilles K, Laird AR, Eickhoff SB (2010). ALE meta-analysis of action observation and imitation in the human brain.. Neuroimage.

[21] Keysers C, Kaas JH, Gazzola V (2010). Somatosensation in social perception.. Nat Rev Neurosci.

[22] Deshpande G, Sathian K, Hu X (2010). Effect of hemodynamic variability on Granger causality analysis of fMRI.. Neuroimage.

[23] Kilner JM, Friston KJ, Frith CD (2007). Predictive coding: an account of the mirror neuron system.. Cogn Process.

[24] Kilner JM, Vargas C, Duval S, Blakemore SJ, Sirigu A (2004). Motor activation prior to observation of a predicted movement.. Nat Neurosci.

[25] Wolpert DM, Doya K, Kawato M (2003). A unifying computational framework for motor control and social interaction.. Philos Trans R Soc Lond B Biol Sci.

[26] Wolpert DM, Ghahramani Z, Jordan MI (1995). An internal model for sensorimotor integration.. Science.

[27] Wolpert DM, Miall RC (1996). Forward Models for Physiological Motor Control.. Neural Netw.

[28] Miall RC (2003). Connecting mirror neurons and forward models.. Neuroreport.

[29] Iacoboni M, Koski LM, Brass M, Bekkering H, Woods RP (2001). Reafferent copies of imitated actions in the right superior temporal cortex.. Proc Natl Acad Sci U S A.

[30] Luppino G, Murata A, Govoni P, Matelli M (1999). Largely segregated parietofrontal connections linking rostral intraparietal cortex (areas AIP and VIP) and the ventral premotor cortex (areas F5 and F4).. Exp Brain Res.

[31] Demiris J, Hayes G, DKaN CL (2002). Imitation as a dual-route process featuring predictive and learning components: a biologically plausible computational model.. Imitation in Animals and Artifacts Cambridge.

[32] Schaal S, Ijspeert A, Billard A (2003). Computational approaches to motor learning by imitation.. Philos Trans R Soc Lond B Biol Sci.

[33] Keller PE, Morganti F, Carassa A, Riva G (2008). Joint Action in Music Performance.. A Cognitive and Social Perspective on the Study of Interactions.

[34] Keller PE, Knoblich G, Repp BH (2007). Pianists duet better when they play with themselves: On the possible role of action simulation in synchronization.. Consciousness and Cognition.

[35] Szymaszek A, Szelag E, Sliwowska M (2006). Auditory perception of temporal order in humans: the effect of age, gender, listener practice and stimulus presentation mode.. Neurosci Lett.

[36] Dunlap K (1910). Reactions on rhythmic stimuli, with attempt to synchronize.. Psychological Review.

[37] Johnson WS (1898). Researches in practice and habit.. Studies from the Yale Psychology Laboratory.

[38] Miyake I (1902). Researches on rhythmic action.. Studies from the Yale Psychology Laboratory.

[39] Repp BH (2005). Sensorimotor synchronization: a review of the tapping literature.. Psychon Bull Rev.

[40] Wolpert DM, Ghahramani Z (2000). Computational principles of movement neuroscience.. Nat Neurosci.

[41] Hietanen JK, Perrett DI (1993). Motion sensitive cells in the macaque superior temporal polysensory area. I. Lack of response to the sight of the animal's own limb movement.. Exp Brain Res.

[42] Hietanen JK, Perrett DI (1996). Motion sensitive cells in the macaque superior temporal polysensory area: response discrimination between self-generated and externally generated pattern motion.. Behav Brain Res.

[43] Blakemore SJ, Wolpert D, Frith C (2000). Why can't you tickle yourself?. Neuroreport.

[44] Blakemore SJ, Frith CD, Wolpert DM (2001). The cerebellum is involved in predicting the sensory consequences of action.. Neuroreport.

[45] Blakemore SJ, Sirigu A (2003). Action prediction in the cerebellum and in the parietal lobe.. Exp Brain Res.

[46] Dum RP, Strick PL (2003). An unfolded map of the cerebellar dentate nucleus and its projections to the cerebral cortex.. J Neurophysiol.

[47] Stein JF, Glickstein M (1992). Role of the cerebellum in visual guidance of movement.. Physiol Rev.

[48] Kawato M, Kuroda T, Imamizu H, Nakano E, Miyauchi S (2003). Internal forward models in the cerebellum: fMRI study on grip force and load force coupling.. Prog Brain Res.

[49] Wolpert DM, Miall RC, Kawato M (1998). Internal models in the cerebellum.. Trends in Cognitive Sciences.

[50] Friston KJ, Penny WD, Glaser DE (2005). Conjunction revisited.. Neuroimage.

[51] Eickhoff SB, Stephan KE, Mohlberg H, Grefkes C, Fink GR (2005). A new SPM toolbox for combining probabilistic cytoarchitectonic maps and functional imaging data.. Neuroimage.

