# Supplementary material for: Granger Causality Mapping during Joint Actions Reveals Evidence for Forward Models That Could Overcome Sensory-Motor Delays
Source: PLoS One. 2010 Oct 21;5(10):e13507. doi: 10.1371/journal.pone.0013507 (PMC2958830; doi:10.1371/journal.pone.0013507)
Supplement: Table S1 — Abbreviations used in the paper together with their meanings. (0.05 MB DOC) [file pone.0013507.s001.doc]

**Table S1.** Abbreviations used in the paper together with their meaning.

|  | *ang* | angle joint action condition |
| --- | --- | --- |
|  | BA44 | Brodmann area 44 |
|  | BA6 | Brodmann area 6 |
|  | BOLD | blood-oxygen-level dependent |
|  | EPI | echo-planer imaging |
|  | *exe* | execution condition |
|  | fMRI | functional magnetic resonance imaging |
|  | GCM | Granger causality mapping |
|  | GLM | general linear model |
|  | Hem | hemisphere |
|  | IFG | inferiror frontal gyrus |
|  | IPL | inferior parietal lobule |
|  | MOG | middle occipital gyrus |
|  | MTG | middle temporal gyrus |
|  | *obs* | observation condition |
|  | PF | parietal area F |
|  | pMNS | putative mirror neuron system, i.e. *obs*>0 AND *exe*>0 |
|  | preCG | precentral gyrus |
|  | RL | red light |
|  | ROI | region of interest |
|  | SB | start button |
|  | SI | primary somatosensory area |
|  | SII | secondary somatosensory area |
|  | SPL | superior parietal lobule |
|  | *str* | straight joint action condition |
|  | Vox | voxel |

All brain areas were labeled using the Anatomy Toolbox for SPM based on probabilistic cytoarchitectonic maps [2].
